# First report on the occurrence of *Rickettsia slovaca *and *Rickettsia raoultii *in *Dermacentor silvarum *in China

**DOI:** 10.1186/1756-3305-5-19

**Published:** 2012-01-19

**Authors:** Zhan-Cheng Tian, Guang-Yuan Liu, Hui Shen, Jun-Ren Xie, Jin Luo, Mei-Yuan Tian

**Affiliations:** 1 State Key Laboratory of Veterinary Etiological Biology, Key Laboratory of Veterinary Parasitology of Gansu Province, Lanzhou Veterinary Research Institute, Chinese Academy of Agricultural Sciences, Lanzhou, Gansu Province 730046, People's Republic of China

## Abstract

**Background:**

Rickettsioses are among both the longest known and most recently recognized infectious diseases. Although new spotted fever group rickettsiae have been isolated in many parts of the world including China, Little is known about the epidemiology of Rickettsia pathogens in ticks from Xinjiang Autonomous Region of China.

**Methods:**

In an attempt to assess the potential risk of *rickettsial *infection after exposure to ticks in Xinjiang Uygur Autonomous Region of China, a total of 200 *Dermacentor silvarum *ticks collected in Xinyuan district were screened by polymerase chain reaction based on the outer membrane protein A gene.

**Results:**

22 of the 200 specimens (11%) were found to be positive by PCR. Phylogenetic analysis of OmpA sequences identified two *rickettsial species, Rickettsia raoultii *(4.5%) and *Rickettsia slovaca *(6.5%).

**Conclusions:**

This study has reported the occurrence of *Rickettsia raoultii *and *Rickettsia slovaca *in Xinjiang Autonomous Region of China and suggests that *Dermacentor silvarum *could be involved in the transmission of rickettsial agents in China. Further studies on the characterization and culture of rickettsial species found in *Dermacentor silvarum *should be performed to further clarify this. Additionally, the screening of human specimens for rickettsial disease in this region will define the incidence of infection.

## Background

Tick-transmitted diseases are a focus of increasing medical interest worldwide. Ticks are the main vectors and reservoirs of rickettsial pathogens responsible for spotted fever. Rickettsioses are among both the longest known and most recently recognized infectious diseases. The clinical features include fever, headache, eruption, and incidental eschar formation at the site of tick bites [1]. The etiological agents belonging to the genus *Rickettsia *are currently divided into two groups: the typhus group and the spotted fever group. The latter group includes an increasing number of newly identified species.

In China, many spotted fever group (SFG) rickettsiae belong to *R. sibirica*, including 2 subspecies, i.e., *R. sibirica sibirica*, the agent of North Asian tick typhus detected in *Dermacentor silvarum *and *D. sinicus *in northern China, and *R. sibirica mongolotimonae*, the agent of lymphangitis-associated rickettsiosis isolated from *Hyalomma asiaticum *in Inner Mongolia [2,3]. *Rickettsia heilongjiangensis*, first isolated from *D. silvarum *ticks in Heilongjiang Province, can cause spotted fever in humans [4,5]. *Rickettsia hulinii *was first isolated from *Haemaphysalis concinna *in Heilongjiang Province, but its pathogenic role in humans has not been demonstrated [6]. However, there is limited information on the epidemiology of rickettsial species in ticks from the Xinjiang Uygur Autonomous Region (XUAR), China, apart from a case report of a SFG rickettsia from a patient in XUAR [7].

In the present study, we assessed the prevalence of rickettsial pathogens in *D. silvarum *from Xinyuan district, XUAR using molecular techniques. Identification and characterization of these circulating agents is crucial for the development of preventive measures in response to the gradually increasing exposure of humans to tick vectors.

## Methods

### Ticks and DNA extraction

A total of 200 adult female ticks were identified as *D. silvarum *based on morphological characteristics [8]. Briefly, the ticks were disinfected in 70% ethanol for 10 min, rinsed with sterilized distilled water, placed in a microtube, and mechanically disrupted with sterile scissors in 50 μl of DNA extraction buffer (10 mM Tris pH 8.0, 2 mM EDTA, 0.1% sodium dodecyl sulfate, and 500 μg of proteinase K per ml). The sample was incubated at 56°C for 4 hr, then boiled at 100°C for 10 min to inactivate the proteinase K. After centrifugation, the supernatant was transferred to a fresh microtube and DNA was purified by extracting twice with an equal volume of phenol-chloroform, precipitated in ethanol and the DNA resuspended in 20 μl elution buffer, which was then stored at -20°C until used.

### PCR amplification and sequence analysis of ompA

PCR reactions were performed using primers Rr190.70p and Rr190.602n (5'-ATGGCGAATATTTCTCCAAAA-3'; 5'-AGTGCAGCATTCGCTCCCCCT-3') designed to amplify the outer membrane protein A (ompA) gene of *rickettsial *species as described previously [9]. Distilled water instead of tick DNA template was used as a negative control. PCR products were purified and sequenced. These were compared with previously published sequences deposited in GenBank using BLAST. Partial ompA sequences of *rickettsial *species were aligned with that of 27 *rickettsial *species by the Clustal W program with default parameter settings (DNAStar version 4.01, Madison, WI, USA). Outer membrane protein P44 from Anaplasma phagocytophila (AF412830) was used as an outlier group in the alignments of nucleotide sequences of ompA. A phylogenetic tree was constructed using the Kimura 2-parameter model and the neighbour-joining algorithm of MEGA 4.0 software [10].

## Results

PCR products of the *rickettsial *ompA gene with expected size (530-533 bp) were amplified from *D. silvarum *ticks. Sequencing data of the 22 positive samples indicated two distinct rickettsial species from the 200 ticks screened. Nine of these were identified as *R. raoutii *and the remaining 13 were *R. slovaca*. Six of the *R. raoutii *samples were 100% identical to each other but exhibited 99.1-99.8% (530/530) variability with the remaining 3 *R. raoutii *samples. However, all 9 samples of *R. raoutii *were 99.8-100% and 99.2-99.4% (511/511) homologous with the *R. raoultii Marne *and *Khabarovsk *strains respectively. Of the 13 *R. slovaca *samples identified, 11 were 100% identical, while the remaining 2 were slightly divergent with a 99.6 and 99.8% (533/533) homology. Collectively all the *R. slovaca *samples were 99.8-100% (533/533) homologous with the *R. slovaca *(HM161798.1) strain. The variability between the two *rickettsial *species identified from the ticks was 5.5-5.8%. All of the unique sequencing data (not including identical sequences) were deposited in GenBank with accession numbers JN400401-JN400407.

**Figure 1:**
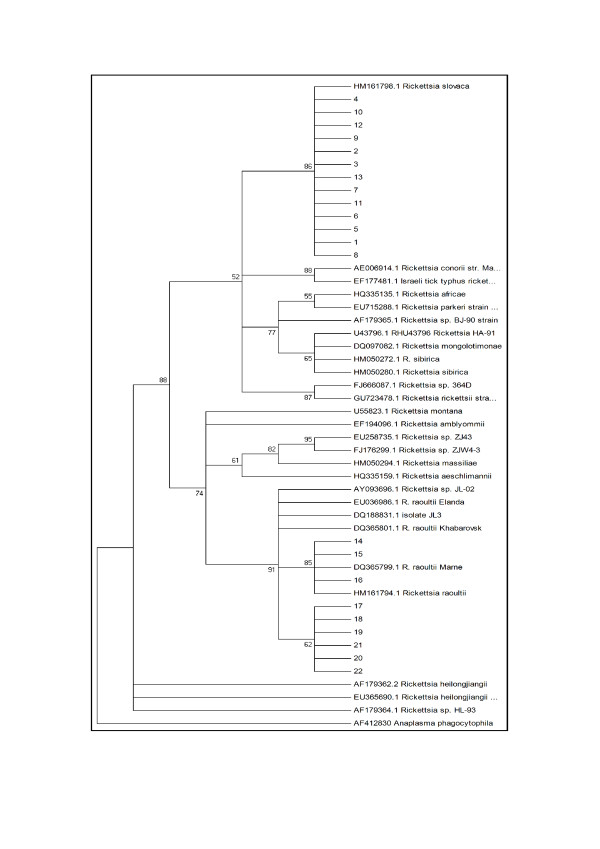
**Constructed phylogenetic tree based on partial ompA gene sequences from *rickettsial *positive tick samples using neighbour-joining method with 1,000 bootstraps and a cut off value of 50%**.

Phylogenetic analysis indicated the formation of 3 clades within the rickettsial neighbour-joining tree (Figure 1). All of *R. slovaca *like samples formed a single clade with *R. slovaca *while the *R. raoutii *like samples formed two clades. One of these clades was closely related to the *R. raoutii *reference strains and the other formed a unique *R. raoutii *clade which is possibly a new strain.

## Discussion

The present study reports for the first time the occurrence of *R. raoultii *and *R. slovaca *in *D. silvarum *ticks in China. *Rickettsia raoultii *has been reported in Dermacentor ticks in Europe and Russia, including strains KhabarovskT, Marne, Shayman, 8/9 Karaganda and Elanda-23/95 [11]. Until the present study *R. raoultii *had not been definitely reported in China and only two ompA gene sequences from two isolates from Jilin province had previously been deposited in GenBank (AY093696.1 and DQ188831.1), however, this was not reported in the literature. The *rickettsial *strains identified in this study were defined by using published phylogenetic classifications of *rickettsial *species [3,6]. PCR and sequencing of *rickettsial *ompA genes, identified *R. raoultii *in 4.5% of *D. silvarum *ticks collected in XUAR. Furthermore, phylogenetic analysis revealed that the positive samples detected formed a distinct clade with *R. raoultii *with a high (91) bootstrap value, which included strains KhabarovskT and Marne. Of the nine *R. raoutii *samples identified in this study, eight were from Xinyuan and one was from Gansu province. Together with the two samples from Jilin province (AY093696.1 and DQ188831.1). This suggests that *R. raoultii *has a geographical spread that encompasses a large area of China.

*Rickettsia slovaca *as a human pathogen [12] was first isolated in 1968 from a *Dermacentor marginatus *tick in Slovakia [13]. Since then, it has been found in both *D. marginatus *and *D. reticulatus *ticks from Western Europe and central Asia [14-18]. The rickettsial disease caused by *R. slovaca *is called tick-borne lymphadenopathy (TIBOLA) or Dermacentor-borne necrosis- erythema-lymphadenopathy [19,20] and its epidemiological pattern and clinical features in patients from France, Hungary and Spain have been investigated [18,21]. It therefore seems to closely follow the distribution of its main host tick, *D. marginatus*, which can be found throughout Europe and as far as the western border of China [15]. However, in the present study, we detected *R. slovaca *by PCR in 6.5% of *D. silvarum *ticks collected from XUAR. The results of the present study identified *R. slovaca *in the *D. silvarum *ticks which could be transmitted to humans and cause disease. Further studies on the characterization and culture of rickettsial endosymbionts found in *D. silvarum *collected in XUAR should be performed.

## Conclusions

This is the first report of *R. raoultii *and *R. slovaca *in China and suggests that *D. silvarum *could be involved in the transmission of *R. slovaca *in China. Further studies on the characterization and culture of rickettsial endosymbionts found in *D. silvarum *from XUAR should be performed.

## Competing interests

The authors declare that they have no competing interests.

## Authors' contributions

LGY and TZC conceived and designed the study, and critically revised the manuscript. TZC, SH and LJ performed the experiments, analysed the data and drafted the manuscript. XJR and TMY helped in study implementation and data collection. All authors read and approved the final manuscript.
